# Longitudinal Patterns of Beverage Intake in Treatment-Seeking Children with Obesity in Eastern NC Using the Validated BEVQ-15

**DOI:** 10.3390/nu15194171

**Published:** 2023-09-27

**Authors:** Zahra Mohseni, Dmitry Tumin, David N. Collier, Natalie Taft, Suzanne Lazorick

**Affiliations:** 1 Brody School of Medicine, East Carolina University, Greenville, NC 27834, USA; zahra.mohseni@atriumhealth.org; 2 Department of Pediatrics, Brody School of Medicine, East Carolina University, Greenville, NC 27834, USA; tumind18@ecu.edu (D.T.); collierd@ecu.edu (D.N.C.); taftn@ecu.edu (N.T.); 3 Department of Public Health, Brody School of Medicine, East Carolina University, 115 Heart Dr, Mailstop 660, Greenville, NC 27834, USA

**Keywords:** child obesity, beverage consumption, sugar-sweetened beverages, child obesity treatment, child obesity intervention

## Abstract

Sugar-sweetened beverage (SSB) consumption remains a major target for interventions to treat severe obesity in children. Understanding how total energy consumption is divided among different types of beverages remains unclear. This study retrospectively examined how the consumption of beverage calories (kcal) from 100% fruit juice and SSBs, and body mass index, assessed as a percent of the 95th sex- and age-specific percentile (%of 95BMI), changed during the treatment of children with obesity aged 2–18 years. Treatment was provided by an integrative multi-disciplinary team, comprising a physician, a dietician/ nutritionist and a behavioralist employing motivational interviewing and a small change approach to promote improved sustainable health habits and induce a net negative energy balance. The sample included 155 patients, with 341 visits. The median age was 11 years, 60% were girls, and there was a median follow-up of 3.1 months. At baseline, the median %of 95BMI was 135 and the median kcal/day intake was 436 from juice and 263 from SSB. For each additional 100 kcal consumed/day from SSB and juice, the %of 95BMI increased by 1.4 percentage points. In the follow-up, each additional month was associated with 7 fewer kcal/day from SSB and juice combined, with a 0.5 percentage point increase in %of 95BMI. Children in this treatment program consumed fewer calories from SSB over time, although the %of 95BMI did not decrease. SSBs other than soda accounted for the majority of beverage kcal intake, therefore potentially providing a targeted direction for interventions.

## 1. Introduction

Severe obesity in childhood is a complex and dynamic disease influenced by a variety of factors, including health behaviors and the societal environment [1]. While the classification of a child’s weight status as obese is based on the child’s Body Mass Index (BMI) meeting or exceeding the threshold of the 95th percentile for age and sex, the severity of obesity in children is further described through an assessment of the BMI relative to the BMI at the 95th percentile. Severe obesity in children is defined as BMI ≥ 120 percent of the 95th percentile for age and sex (%of 95BMI). The prevalence of severe obesity among US children varies from 1.8% in 2–5-year-old children to 9.5% in 16–19-year-olds [2].

Although the overall consumption of sugar-sweetened beverages (SSB) has declined among children in the last decade [3] and the prevalence of heavy SSB intake (>500 kcal/day) has decreased from 11% to 3% among children in the general population [4], decreasing excess caloric intake from sugar-sweetened beverages (SSB) in individual children undergoing treatment for severe obesity is a major target for interventions [5]. Recent studies have shown that SSB consumption promotes a higher BMI in children [6], and that children receiving interventions to reduce SSB consumption experience lower rates of BMI gain [6], highlighting the importance of SSBs in obesity treatment. As decreases in SSB consumption may be offset by increasing the consumption of food or other energy-dense beverages, a narrow focus on SSBs may potentially limit the efficacy of treatment. A way for clinicians to gain insight into the beverages chosen by patients and to better understand how energy consumed from beverage intake changes over the course of treatment is to quantify not only the total energy consumed from beverages, but also the energy consumed from various subtypes of calorie-dense beverages.

Few studies have followed the trajectory of beverage consumption in pediatric patients undergoing treatment for weight management using a detailed tool that captures different beverage subtypes [7]. Studies have assessed SSB intake using a variety of methods, such as a food frequency questionnaire (FFQ) [8,9], 24 h dietary recall [10,11], and lifestyle questionnaires [12,13]. However, these methods rely on estimated beverage serving size, without assessing the specific calories or volume from beverage subtypes. In addition, most studies have not examined patterns of beverage intake over time. In this study, we used an adapted Beverage Intake Questionnaire (BEVQ-15) [14] to evaluate the caloric intake from SSBs and juice at baseline in 625 children present at a pediatric weight management clinic, with the primary aim of determining how the consumption of SSBs and juice changed during the early course of treatment in a subsample of these children with a longitudinal follow-up. Our secondary aims were to evaluate the relationship between beverage consumption and BMI during follow-up, demographic factors, and health behaviors at baseline, and to determine if the pattern of beverage consumption was associated with BMI change during the course of treatment.

## 2. Material and Methods

### 2.1. Study Setting

The pediatric patients included in this study sought treatment for obesity at a Comprehensive Healthy Weight Clinic (CHWC) associated with an academic medical center in the southeast United States. The CHWC is a clinic that offers comprehensive consultations and treatment for children between the ages of 6 months and 19 years who were referred by their health care providers, including primary care and specialists, concerning their weight. The CHWC serves a 42-county region that mostly comprises poor, rural counties. In this clinic, using a family and strengths-based approach the initial consultation encompasses assessment for underlying causes and consequences of excess adiposity, energy imbalance, and strategies for appropriate health behavior changes. Initial assessment includes evaluations by a pediatrician and registered dietician who specialize in childhood obesity and lab work for common comorbidities (e.g., hyperlipidemia, insulin resistance, diabetes, non-alcoholic fatty liver disease). Using motivational interviewing and a small change model, treatment emphasizes setting personalized goals for health behavior changes to achieve improved healthy energy balance including sleep, nutrition, sedentary/active time, and relationship with food. Pharmacotherapy for weight loss, when used, is typically not initiated in the first or second visits. The intended, optimal follow up schedule includes virtual visits with the dietician alternating with in-clinic medical appointments with the physician, at approximately 3–4 week intervals. However, due to scheduling, the distance to clinic, school, and work constraints and other challenges, the frequency and interval of the follow-up visits can vary considerably.

### 2.2. Inclusion and Exclusion Criteria

All data for this study were obtained from a retrospective review of patient charts in the electronic medical record (EMR). Patients aged 2–18 years were included in the baseline study if they completed a first visit between January 2017 and December 2019 and had data on energy intake from beverages (described in detail below). Patients were included in the longitudinal cohort if they also had at least one in-clinic follow-up visit, with the data on energy intake from beverages obtained at the follow-up visit. Unless otherwise noted, each completed visit was considered as a separate observation. The data from follow-up visits were collected throughout July 2021. For multivariable analysis, we excluded cases with missing data on study covariates, as described further below.

### 2.3. Outcomes

The primary outcome of this study was the total energy (measured in kilocalories, kcal) consumed from 100% fruit juice and SSBs. This was ascertained using the BEVQ-15, a quantitative questionnaire validated in adults to assess habitual beverage consumption [14,15,16], but modified for use in children, as previously described [17]. The BEVQ-15 assessment includes questions about intake frequency, per week or per day, and the quantity of water, 100% fruit juice, sweetened juice drinks, whole milk, reduced-fat milk, low-fat milk, flavored milk, carbonated sweetened drinks, diet carbonated drinks, sweet tea, tea with or without sweetener, and sport drinks. For this study, SSBs included soda, sweetened juice or fruit flavored drinks, sports drinks, energy drinks, and coffee or tea sweetened with sugar. The BEVQ-15 is routinely administered by the dietitian at the initial visit, and at select follow-up visits depending on clinical need. While there is no systematic process to determine if the BEVQ-15 is administered at follow-up visits, it is generally not conducted if a patient reports no further changes to beverage intake, or if the calories from beverage intake are deemed a less important contributing factor in the patient’s treatment. Conversely, the use of the tool is emphasized especially when unexpected weight gain occurs with no clear explanation, or when the patient reports a low intake of SSBs in the setting of weight gain. In the primary analysis, we examined the beverage calories from all SSBs and 100% fruit juice. In the secondary analysis, we examined the beverage calories from categories of interest of beverage subtypes as follows: (1) soda, (2) 100% fruit juice, and (3) other sugar-sweetened beverages. Additionally, we examined the BMI at each visit, expressed as a percent of the 95th percentile for sex and age (%of 95BMI) [18].

### 2.4. Independent Variables

The primary independent variable was time since treatment initiation, in months (0 = baseline visit). Additional independent variables extracted from the baseline visit included the patient’s sex, age in months, race/ethnicity, insurance coverage as a proxy for socioeconomic status and healthcare access (Medicaid, the United States federal insurance program for low-income children vs. any other), and additional socioeconomic characteristics, including family composition (living with 2 parents; living with mother only; any other family structure), and whether the child had experienced food insecurity within the past 12 months (assessed by validated questions as part of an intake questionnaire: family worried that food would run out, or food did not last and family did not have money to purchase more) [19]. Dietary behaviors at baseline included the number of meals eaten out per week, and the number of days/week the child ate breakfast (recoded to the midpoint, if the response was documented as a range).

### 2.5. Data Analysis

Study variables were summarized using medians and interquartile ranges (IQR), or counts and percentages, as appropriate. At the baseline visit and at the most recent follow-up, the energy intake from beverages (total SSBs and juice, and by beverage type) was correlated with the %of 95BMI using Spearman correlation coefficients. Change over time in energy intake was modeled using quantile regression with patient-level cluster-robust standard errors [20], where each visit was treated as a separate observation, and independent variables included time on treatment (in months) and baseline covariates. Similarly, change over time in BMI was modeled using quantile regression with cluster-robust standard errors, controlling for either total energy intake from beverages, or energy intake from each specific beverage type, at the time of the baseline visit. In the initial sample (before excluding patients with no follow-up visits), we analyzed each outcome (energy intake from beverages and the current %of 95BMI) using the quantile regression of cross-sectional data from the baseline visits only. Data analysis was performed in Stata/SE 16.1 (StataCorp, LP: College Station, TX, USA), and *p* < 0.05 was considered statistically significant.

## 3. Results

We identified 625 patients aged 2–18 years who completed the BEVQ-15 and had their BMI recorded at the initial visit, and these were included in the baseline analysis. After excluding 467 patients who did not complete a follow-up visit and 3 patients who did not complete the BEVQ-15 at any follow-up visit, we retained 155 patients and 341 visits for longitudinal analysis. Of this group, 128 patients contributed 2 visits, 24 contributed 3 visits, 2 patients contributed 4 visits each, and 1 patient contributed 5 visits to the longitudinal dataset. Across all 155 patients, the median time from baseline to the last available follow-up was 3.1 months (IQR: 1.6, 9.0). At baseline, the overall median age was 13 years (IQR 10, 14) and the median age was 11 years for those with follow-up (IQR: 9, 14). Overall, there were 356 female and 268 male patients, with 93 female and 62 male patients in the longitudinal subset. At baseline, for all 625 patients, the median %of 95BMI was 131 (IQR: 115, 148). The baseline %of 95BMI for the longitudinal cohort was 135 (IQR: 121, 152), as compared to 138 (IQR: 121, 155) at the most recent follow-up. Additional participant characteristics at the baseline visit are summarized in Table 1.

The energy intake from beverages is summarized for the baseline and most recent follow-up visit in Table 2. The median caloric intake from SSBs and 100% juice at baseline was 270 kcal/day (IQR: 126, 554) in the overall sample and 436 kcal/day (IQR: 248, 765) in the longitudinal cohort, whereas the median caloric intake specifically from SSBs was 172 kcal/day (IQR: 68, 372) and 263 kcal/day (IQR: 130, 602) for the overall sample and longitudinal cohort, respectively. At the most recent follow-up visit, the median caloric intake from SSBs and 100% fruit juice combined decreased to 133 kcal/day (IQR: 72, 237), and the median caloric intake from SSBs decreased to 78 kcal/day (IQR: 31, 153) in the longitudinal cohort. At baseline, a higher caloric intake from SSBs was weakly correlated with a greater %of 95BMI in the final sample (N = 155, rho = 0.18; *p* = 0.023). Likewise, the combined caloric intake from SSBs and 100% juice was weakly correlated at the study baseline with a greater %of 95BMI in the final sample (N = 155, rho = 0.16; *p* = 0.046). These correlations were similar when examining the most recent follow-up visit for each patient (SSB rho = 0.19, *p* = 0.020; SSB + 100% fruit juice rho = 0.15, *p* = 0.069).

A multivariable analysis of the total beverage calories in the final (longitudinal) sample is summarized in Table 3, including 147 patients and 321 observations (visits) with complete data. For each additional month that passed since the baseline visit, patients consumed seven fewer calories per day from SSBs and 100% fruit juice combined (coefficient: −6.8; 95% confidence interval [CI]: −11.6, −2.0; *p* = 0.005). Regarding our analysis of beverage subtypes, we found decreases specifically for soda (−0.7; 95% CI −1.2, −0.1; *p* = 0.014) and non-soda SSBs (−3.6; 95% CI −6.2, −1.0; *p* = 0.008), but not for 100% fruit juice (Tables S1–S3). In addition, Table 3 showed that Hispanic or Latino patients tended to consume fewer calories per day from SSBs and 100% fruit juice than non-Hispanic Black patients (−123.8; 95% CI −225.9, −21.6; *p* = 0.018). Patients who had Medicaid insurance consumed more calories from SSBs and 100% fruit juice per day than those with other types of insurance (108.8; 95% CI 12.5, 205.0; *p* = 0.027). Lastly, children who ate meals out on more days per week tended to consume more calories from SSBs and 100% fruit juice (52.2; 95% CI 18.4, 86; *p* = 0.003). Neither sex nor family composition, food insecurity, or breakfast consumption influenced caloric intake for SSBs and 100% fruit juice.

A multivariable analysis of the %of 95BMI in the final (longitudinal) sample is shown in Table 4. For each additional month on treatment, the %of 95BMI increased by 0.5 points (coefficient: 0.5; 95% confidence interval [CI]: 0.1, 1.0; *p* = 0.012). In addition, for each additional 100 calories consumed per day from SSBs and 100% fruit juice, the %of 95BMI increased by 1.4 points (coefficient: 1.4; 95% confidence interval [CI]: 0.8, 2.0; *p* < 0.001). Table 4 also showed that non-Hispanic White patients had a lower %of 95BMI compared to non-Hispanic Black patients. Upon analysis of beverage subtypes, the number of calories consumed specifically from soda was associated with a higher %of 95BMI (coefficient per 100 kcal increase in calories consumed from soda: 2.7; 95% CI: 0.4, 5.1; *p* = 0.022), but this outcome was not associated with the number of calories consumed from 100% juice or SSBs other than soda (Table S4).

All multivariable analyses were repeated for the initial (cross-sectional) sample, including patients with complete data from the baseline visit (Tables S5–S10). Consistent with Table 3, the energy consumption from SSBs and 100% fruit juice was substantially lower in White and Hispanic patients than in Black patients, and higher with more meals eaten out (Table S5). The energy intake from beverages was not associated with the %of 95BMI, whether grouping calories from all beverage types together (Table S9) or analyzing calories from soda, juice, and other beverages separately (Table S10).

## 4. Discussion

Sugar-sweetened beverage consumption remains a major target for interventions to treat severe childhood obesity [21,22,23]. Although SSB consumption has declined among children in the past decade, the prevalence of obesity in children continues to rise [3,24]. Understanding how total energy consumption from beverages changes over the course of obesity treatment offers potential to inform how current interventions are affecting energy-dense beverage choices. While we cannot assume causation, this study found that in one clinic setting, the longer children are in treatment for weight management, the fewer calories they consume from SSBs and 100% fruit juice, with a decline on average of about 7 kcal/day for every additional month in treatment. It is notable that beverages other than soda and 100% fruit juice accounted for the most drink calories consumed in this sample. Public insurance status and the frequency of eating meals out were both associated with a higher caloric intake from beverages (total SSBs + 100% juice), but other social factors, including family composition, food insecurity, and the frequency of eating breakfast, were not associated with caloric intake from these beverages.

Paradoxically, the decrease in energy intake from beverages was not correlated with improved weight status over the course of treatment. Although children who consume more calories from beverages tend to have a higher %of 95BMI and drink fewer calories from beverages while progressing in treatment, no individual beverage subtype was significantly associated with a decrease in BMI in our analysis during the follow-up period.

A high consumption of SSBs is associated with excessive weight gain and the development of overweight in the pediatric population [25,26,27]. SSB consumption contributes to a positive energy balance due to these drinks’ high energy density and low nutritional value [25]. Although population-level data suggest that SSB consumption has decreased throughout all pediatric age groups, the consumption of SSBs in the pediatric population remains high [28]. Studies have focused on targeting SSB intake in the general pediatric population, while children suffering from obesity are at the highest risk of developing further complications such as type 2 diabetes or cardiovascular disease [29]. With intervention, children with obesity have the opportunity to modify harmful behaviors, such as the consumption of high-energy beverages [30]. This study analyzed the trajectory of beverage consumption with a detailed tool that allowed a breakdown of beverage subtype and serving sizes as potential treatment targets. In addition, the beverage subtype analyses revealed that beverages other than soda and 100% fruit juice were responsible for most of the kcal consumption from SSBs. Following children as they progress through obesity treatment provides an opportunity for clinicians to understand patterns in the patient’s dietary habits that might not have been apparent on initial visits with a snapshot of their beverage intake. In addition, it can provide an insight into the decision-making process behind SSB consumption in the adolescent population [31], which can influence the approach providers take when providing personalized obesity interventions in certain age groups.

In this study, the consumption of all subtypes of SSB decreased over the course of treatment, but this decrease did not correlate with a decrease in BMI measures. Although this finding was unexpected given the decrease in SSB consumption, it can provide useful information regarding the relationship between weight status and beverage intake, such as the length of intervention that is needed to see a change in weight status. In addition, some studies have suggested that SSBs need to be targeted in the prevention of excessive weight gain in children [27], but this study provides evidence identifying possible gaps in interventions for weight reduction. For example, if interventions target reducing soda and 100% fruit juice, our data suggest that focus is also needed, and may be more important, on the other beverage types that are consumed at higher levels in some children with severe obesity.

There are several limitations of this study. The data were from a single site, which can contribute to a lack of diversity in the patient sample, represents one treatment model, and may therefore not be generalizable. The study is observational and so our findings are associative and cannot be interpreted to imply causation. In addition, there were high attrition rates beyond levels typical of obesity treatment settings, possibly due to the turnover of clinic administrative staff during this time and the challenges faced by clinics and patients during the COVID-19 pandemic that overlapped with the follow-up period of the study. Since not every patient was routinely re-evaluated for drink intake with the quantitative tool, there was a potential selection bias regarding the low number of re-surveyed patients. Patients who were re-evaluated may have been selected in part based on the lack of success they had with obesity treatment; therefore, those who were least successful were likely to have been re-evaluated more often. To prevent this selection bias, a universal re-evaluation of all patients regardless of weight or BMI change should be considered. The timeframe of the study might not have been long enough to capture the changes in BMI that would be expected to accompany a decrease in beverage consumption. Finally, we did not assess the contribution of physical activity or other dietary changes which may have impacted BMI measures.

This study suggests that in the first few months of treatment for children with severe obesity, decreased SSB consumption may not correlate to a significant change in BMI measures. This provides some insight into the effectiveness of the interventions used during the treatment sessions for this population. Although beverage consumption may be changing in the desired direction, more time is needed to assess changes in BMI measures. In addition, beverage consumption is a fraction of the calories consumed in a given day, therefore providing only a snapshot of the patterns of intake in children with obesity. The BEVQ-15 provides a detailed and objective measurement of the calories consumed from beverages; pairing this questionnaire with other forms of diet assessments and assessment of physical activity and sleep can provide greater benefit for intervention. Longitudinal patterns of intake can aid treatment teams with tailoring education and interventions for children to make sustainable changes in their health behaviors and weight status.

## 5. Conclusions

Children with obesity in this treatment setting at baseline consumed substantial calories from beverages, with the majority from beverage types other than soda or 100% fruit juices. While calories consumed from beverages decreased during treatment, this was not associated with improved weight status in the same time frame. The BEVQ-15 can be a useful tool for better understanding the contribution of beverage calories, the types of beverages consumed, and the changes over time to target interventions for children with obesity. 

## Figures and Tables

**Table 1:** Patient characteristics at baseline.

Variable	Initial Sample (N = 625)	Final Sample (N = 155)
	Median (IQR) or N (%)	Median (IQR) or N (%)
Sex ^a^		
Female	356 (57%)	93 (60%)
Male	268 (43%)	62 (40%)
Age (years) ^a^	13 (10, 14)	11 (9, 14)
Race/ethnicity		
Non-Hispanic Black	345 (55%)	99 (64%)
Non-Hispanic White	114 (18%)	13 (8%)
Hispanic or Latino	128 (20%)	35 (23%)
Other	38 (6%)	8 (5%)
Medicaid insurance	492 (79%)	135 (87%)
Family composition ^b,c^		
Two parents	323 (53%)	72 (47%)
Mother only	175 (29%)	50 (33%)
Other	115 (19%)	30 (20%)
Food insecurity ^b,d^	173 (29%)	53 (35%)
Meals out (days/week) ^e,f^	2.5 (0.5, 2.5)	2.5 (0.5, 2.5)
Breakfast (days/week) ^g,h^	7 (2, 7)	7 (2, 7)
BMI as percent of 95th percentile	131 (115, 148)	135 (121, 152)

BMI, body mass index; IQR, interquartile range. ^a^ Data missing in 1 case in initial sample. ^b^ Data missing in 3 cases in final sample. ^c^ Data missing in 12 cases in initial sample. ^d^ Data missing in 18 cases in initial sample. ^e^ Data missing in 5 cases in final sample. ^f^ Data missing in 15 cases in initial sample. ^g^ Data missing in 6 cases in final sample. ^h^ Data missing in 23 cases in initial sample.

**Table 2:** Energy intake from beverages at baseline and most recent visit.

	Initial Sample (N = 625)	Final Sample (N = 155)	
Beverage type (kcal/day)	Baseline visit	Baseline visit	Most recent visit
	Median (IQR)	Median (IQR)	Median (IQR)
SSB + 100% fruit juice	270 (126, 554)	436 (248, 765)	133 (72, 237)
SSBs only	172 (68, 372)	263 (130, 602)	78 (31, 153)
Soda	27 (0, 53)	40 (0, 93)	0 (0, 27)
Other SSBs	124 (44, 285)	190 (73, 403)	53 (18, 118)
100% fruit juice	53 (0, 124)	71 (18, 212)	35 (0, 71)

IQR, interquartile range; SSB, sugar-sweetened beverage.

**Table 3:** Multivariable quantile regression of energy intake from sugar-sweetened beverages and 100% fruit juice (kcal/day; N = 321 visits).

Variable	Coefficient	95% CI	*p*
Time since baseline (months)	−6.8	−11.6, −2.0	0.005
Sex			
Female	Ref.		
Male	29.9	−46.2, 106.0	0.440
Age (years)	4.2	−7.6, 16.0	0.483
Race/ethnicity			
Non-Hispanic Black	Ref.		
Non-Hispanic White	−66.0	−218.4, 86.3	0.394
Hispanic or Latino	−123.8	−225.9, −21.6	0.018
Other	−80.4	−208.7, 47.9	0.219
Medicaid insurance	108.8	12.5, 205.0	0.027
Family composition			
Two parents	Ref.		
Mother only	32.4	−62.4, 127.1	0.502
Other	−47.6	−162.3, 67.0	0.414
Food insecurity	−29.7	−112.5, 53.1	0.481
Meals out (days/week)	52.2	18.4, 86.0	0.003
Breakfast (days/week)	−10.7	−24.7, 3.3	0.134

CI, confidence interval; Ref., reference category.

**Table 4:** Multivariable quantile regression of percent over 95th percentile of body mass index (N = 321 visits).

Variable	Coefficient	95% CI	*p*
Time since baseline (months)	0.5	0.1, 1.0	0.012
Calories from SSB + 100% fruit juice (100 kcal/day)	1.4	0.8, 2.0	<0.001
Sex			
Female	Ref.		
Male	−3.2	−12.1, 5.6	0.471
Age (years)	−0.2	−1.9, 1.4	0.784
Race/ethnicity			
Non-Hispanic Black	Ref.		
Non-Hispanic White	−16.3	−31.6, −1.0	0.036
Hispanic or Latino	−3.8	−17.3, 9.8	0.587
Other	2.5	−22.2, 27.3	0.840
Medicaid insurance	−4.6	−20.7, 11.4	0.571
Family composition			
Two parents	Ref.		
Mother only	0.7	−11.3, 12.7	0.913
Other	10.3	−1.7, 22.4	0.091
Food insecurity	6.3	−3.6, 16.3	0.212
Meals out (days/week)	−0.8	−2.9, 1.3	0.459
Breakfast (days/week)	−1.5	−4.3, 1.3	0.302

CI, confidence interval; Ref., reference category; SSB, sugar-sweetened beverages.

## Data Availability

Data are not publicly available to protect patient confidentiality. Deidentified data required to replicate study results may be available upon request from the corresponding author.

## Supplementary Materials

The following supporting information can be downloaded at: https://www.mdpi.com/article/10.3390/nu15194171/s1. Table S1: Multivariable quantile regression of energy intake from Soda (kcal/day; N = 321 visits); Table S2: Multivariable quantile regression of energy intake from Juice (kcal/day; N = 321 visits); Table S3: Multivariable quantile regression of energy intake from non-soda SSB (kcal/day; N = 321 visits); Table S4: Multivariable quantile regression of percent of 95th percentile body mass index (N-321 visits); Table S5: Multivariable cross-sectional quantile regression of energy intake from sugar sweetened beverages and 100% fruit juice (kcal/day; N = 590 patients); Table S6: Multivariable cross-sectional quantile regression of energy intake from Soda (kcal/day; N = 591 patients); Table S7: Multivariable cross-sectional quantile regression of energy intake from 100% fruit juice (kcal/day; N = 591 patients); Table S8: Multivariable cross-sectional quantile regression of energy intake from non-soda SSB (kcal/day; N = 590 patients); Table S9: Multivariable cross-sectional quantile regression of percent of 95th percentile of body mass index (N = 590 patients; all beverage types together); Table S10: Multivariable cross-sectional quantile regression of percent of 95th percentile of body mass index (N = 590 patients; beverage types separated).
