# Supplementary material for: Longitudinal Patterns of Beverage Intake in Treatment-Seeking Children with Obesity in Eastern NC Using the Validated BEVQ-15
Source: Nutrients. 2023 Sep 27;15(19):4171. doi: 10.3390/nu15194171 (PMC10648911; doi:10.3390/nu15194171)
Supplement: Supplementary file 1 [file nutrients-15-04171-s001.zip › nutrients-2590742-supplementary.pdf]

nutrients

MDPI

Table S1. Multivariable quantile regression of energy intake from Soda (kcal/day; N=321 visits).

Variable	Coefficient	95% CI	P
Time since baseline (months)	-0.7	-1.2, -0.1	0.014
Sex			
Female	Ref.		
Male	-0.5	-12.5, 11.5	0.938
Age (years)	1.3	-0.8, 3.5	0.220
Race/ethnicity			
Non-Hispanic Black	Ref.		
Non-Hispanic White	20.1	-14.1, 55.9	0.241
Hispanic or Latino	0.8	-13.1, 14.7	0.910
Other	-4.5	-24.6, 15.5	0.656
Medicaid insurance	15.3	-1.0, 31.7	0.066
Family composition			
Two parents	Ref.		
Mother only	-1.8	-14.4, 10.9	0.783
Other	-3.4	-24.8, 18.1	0.757
Food insecurity	-1.5	-15.5, 12.4	0.830
Meals out (days/week)	3.3	-1.6, 8.2	0.182
Breakfast (days/week)	0.4	-1.9, 2.7	0.730

CI, confidence interval; Ref., reference category.

Table S2. Multivariable quantile regression of energy intake from 100% Juice (kcal/day; N=321 visits).

Variable	Coefficient	95% CI	P
Time since baseline (months)	-0.4	-1.5, 0.6	0.428
Sex			
Female	Ref.		
Male	8.1	-14.6, 30.7	0.484
Age (years)	-3.4	-7.2, 0.5	0.084
Race/ethnicity			
Non-Hispanic Black	Ref.		
Non-Hispanic White	-21.6	-554.8, 11.6	0.202
Hispanic or Latino	-38.0	-59.1, -17.0	<0.001
Other	-16.5	-62.3, 29.4	0.480
Medicaid insurance	4.6	-28.2, 37.5	0.782
Family composition			
Two parents	Ref.		
Mother only	9.1	-14.3, 32.6	0.444
Other	-3.7	-41.4, 33.9	0.845
Food insecurity	-23.0	-46.1, 0.1	0.051
Meals out (days/week)	-3.5	-9.7, 2.7	0.265
Breakfast (days/week)	3.4	0.5, 6.2	0.021

CI, confidence interval; Ref., reference category.

Table S3. Multivariable quantile regression of energy intake from non-soda SSB (kcal/day; N=321 visits).

Variable	Coefficient	95% CI	P
Time since baseline (months)	-3.6	-6.2, -1.0	0.008
Sex			
Female	Ref.		
Male	1.3	-50.6, 53.3	0.960
Age (years)	6.3	1.0, 13.5	0.089
Race/ethnicity			
Non-Hispanic Black	Ref.		
Non-Hispanic White	-81.0	-160.4, -1.5	0.046
Hispanic or Latino	-66.8	-137.9, 4.4	0.066
Other	-71.1	-161.2, 19.0	0.121
Medicaid insurance	28.3	-41.4, 98.1	0.425
Family composition			
Two parents	Ref.		
Mother only	-20.6	-81.4, 40.2	0.505
Other	-56.9	-133.1, 19.4	0.143
Food insecurity	10.0	-40.4, 60.4	0.697
Meals out (days/week)	29.9	6.3, 53.4	0.013
Breakfast (days/week)	-7.1	-16.9, 2.7	0.157

CI, confidence interval; Ref., reference category.

Table S4. Multivariable quantile regression of percent of 95th percentile of body mass index (N=321 visits).

**Variable**	**Coefficient**	**95% CI**	**P**
Time since baseline (months)	0.3	0.0, 0.7	0.064
Calories from soda (100 kcal/day)	2.7	0.4, 5.1	0.022
Calories from 100% juice (100 kcal/day)	0.9	-1.2, 3.0	0.392
Calories from SSB other than soda (100 kcal/day)	0.1	-1.7, 2.0	0.876
Sex			
Female	Ref.		
Male	-3.7	-12.4, 5.0	0.400
Age (years)	-0.4	-1.9, 1.2	0.635
Race/ethnicity			
Non-Hispanic Black	Ref.		
Non-Hispanic White	-16.4	-32.8, 0.1	0.052
Hispanic or Latino	-1.9	-14.0, 10.1	0.750
Other	0.7	-19.3, 20.8	0.941
Medicaid insurance	-4.9	-21.3, 11.6	0.561
Family composition			
Two parents	Ref.		
Mother only	1.1	-11.0, 13.2	0.857
Other	10.5	-2.8, 23.7	0.121
Food insecurity	6.0	-3.9, 15.8	0.234
Meals out (days/week)	-0.4	-3.4, 2.7	0.800
Breakfast (days/week)	-1.4	-4.0, 1.3	0.302

CI, confidence interval; Ref., reference category; SSB, sugar sweetened beverages

Table S5. Multivariable cross-sectional quantile regression of energy intake from sugar-sweetened beverages and 100% fruit juice (kcal/day; N=590 patients).

Variable	Coefficient	95% CI	P
Sex			
Female	Ref.		
Male	-12.0	-72.4, 48.5	0.698
Age (years)	-1.9	-11.2, 7.3	0.679
Race/ethnicity			
Non-Hispanic Black	Ref.		
Non-Hispanic White	-176.1	-258.4, -93.9	0.000
Hispanic or Latino	-128.2	-208.7, -47.7	0.002
Other	-.31	-135.6, 135.0	0.996
Medicaid insurance	58.3	-20.1, 136.7	0.145
Family composition			
Two parents	Ref.		
Mother only	2.4	-70.0, 74.8	0.948
Other	49.0	-32.8, 130.9	0.239
Food insecurity	51.0	-15.9, 117.9	0.135
Meals out (days/week)	61.4	39.0, 83.9	0.000
Breakfast (days/week)	.36	-11.2, 12.0	0.951

CI, confidence interval; Ref., reference category

Table S6. Multivariable cross-sectional quantile regression of energy intake from Soda (kcal/day; N=591 patients).

**Variable**	**Coefficient**	**95% CI**	**P**
Sex			
Female	Ref.		
Male	-4.1	-15.8, 7.5	0.488
Age (years)	0.1	-1.7, 1.9	0.919
Race/ethnicity			
Non-Hispanic Black	Ref.		
Non-Hispanic White	-5.2	-21.1, 1.07	0.519
Hispanic or Latino	-6.1	-21.7, 9.4	0.439
Other	5.6	-20.5, 31.7	0.673
Medicaid insurance	11.7	-3.3, 26.8	0.126
Family composition			
Two parents	Ref.		
Mother only	-5.9	-19.8, 8.1	0.407
Other	8.2	-7.6, 24.0	0.307
Food insecurity	19.0	6.1, 31.9	0.004
Meals out (days/week)	5.2	0.9, 9.6	0.018
Breakfast (days/week)	-2.1	-4.3, 0.2	0.070

CI, confidence interval; Ref., reference category

Table S7. Multivariable cross-sectional quantile regression of energy intake from 100% Juice (kcal/day; N=591 patients).

Variable	Coefficient	95% CI	P
Sex			
Female	Ref.		
Male	11.0	-3.8, 25.7	0.145
Age (years)	-3.7	-5.9, -1.4	0.002
Race/ethnicity			
Non-Hispanic Black	Ref.		
Non-Hispanic White	-42.1	-62.2, -22.1	<0.001
Hispanic or Latino	-32.9	-52.5, -13.3	0.001
Other	-17.2	-50.1, 15.8	0.306
Medicaid insurance	12.2	-6.8, 31.2	0.208
Family composition			
Two parents	Ref.		
Mother only	1.8	-15.8, 19.4	0.839
Other	11.0	-9.0, 30.9	0.281
Food insecurity	-16.4	-32.7, -0.2	0.048
Meals out (days/week)	3.3	-2.1, 8.8	0.234
Breakfast (days/week)	3.7	0.8, 6.4	0.011

CI, confidence interval; Ref., reference category

Table S8. Multivariable cross-sectional quantile regression of energy intake from non-soda SSB (kcal/day; N=590 patients).

Variable	Coefficient	95% CI	P
Sex			
Female	Ref.		
Male	3.3	-46.8, 53.3	0.898
Age (years)	1.1	-6.6, 8.7	0.784
Race/ethnicity			
Non-Hispanic Black	Ref.		
Non-Hispanic White	-93.9	-162.0, -25.8	0.007
Hispanic or Latino	-86.5	-153.2, -19.9	0.011
Other	-33.1	-145.1, 80.0	0.562
Medicaid insurance	41.0	-24.0, 105.9	0.216
Family composition			
Two parents	Ref.		
Mother only	-28.0	-88.0, 31.9	0.359
Other	6.6	-61.1, 74.4	0.848
Food insecurity	39.9	-15.5, 95.3	0.158
Meals out (days/week)	51.7	33.1, 70.3	0.000
Breakfast (days/week)	-8.6	-18.2, 1.0	0.078

CI, confidence interval; Ref., reference category

Table S9. Multivariable cross-sectional quantile regression of percent of 95th percentile of body mass index (N=590 patients; all beverage types together).

Variable	Coefficient	95% CI	P
Calories from SSB + 100% fruit juice (100 kcal/day)	-0.1	-0.8, 0.6	0.767
Sex			
Female	Ref.		
Male	0.7	-4.5, 5.9	0.794
Age (years)	-0.7	-1.5, 0.1	0.096
Race/ethnicity			
Non-Hispanic Black	Ref.		
Non-Hispanic White	-4.0	-11.1, 3.1	0.271
Hispanic or Latino	-10.9	-17.8, -3.9	0.002
Other	-6.4	-17.9, 5.2	0.281
Medicaid insurance	-4.1	-10.8, 2.7	0.234
Family composition			
Two parents	Ref.		
Mother only	0.6	-5.6, 6.8	0.857
Other	-0.8	-7.8, 6.2	0.824
Food insecurity	4.8	-0.9, 10.5	0.100
Meals out (days/week)	0.3	-1.7, 2.3	0.761
Breakfast (days/week)	-1.6	-2.6, -0.6	0.002

CI, confidence interval; Ref., reference category; SSB, sugar sweetened beverages

Table S10. Multivariable cross-sectional quantile regression of percent of 95th percentile of body mass index (N=590 patients; beverage types separate).

**Variable**	**Coefficient**	**95% CI**	**P**
Calories from soda (100 kcal/day)	0.3	-1.6, 2.1	0.771
Calories from 100% juice (100 kcal/day)	-0.8	-2.6, 0.9	0.341
Calories from SSB other than soda (100 kcal/day)	0.0	-1.2, 1.7	0.982
Sex			
Female	Ref.		
Male	1.3	-4.2, 6.7	0.648
Age (years)	-0.7	-1.5, 0.2	0.108
Race/ethnicity			
Non-Hispanic Black	Ref.		
Non-Hispanic White	-3.9	-11.4, 3.6	0.304
Hispanic or Latino	-10.7	-18.0, -3.4	0.004
Other	-7.0	-19.1, 5.1	0.257
Medicaid insurance	-4.0	-11.1, 3.0	0.262
Family composition			
Two parents	Ref.		
Mother only	0.5	-6.0, 7.0	0.883
Other	-0.8	-8.2, 6.5	0.822
Food insecurity	5.8	-0.2, 11.8	0.056
Meals out (days/week)	0.2	-1.9, 2.3	0.873
Breakfast (days/week)	-1.5	-2.6, -0.5	0.005

CI, confidence interval; Ref., reference category; SSB, sugar sweetened beverages
